# Six new species of the genus *Laena* Dejean from China (Coleoptera, Tenebrionidae, Lagriinae)

**DOI:** 10.3897/zookeys.177.2426

**Published:** 2012-03-23

**Authors:** Zhao Xiao-Lin, Ren Guo-Dong

**Affiliations:** 1 College of Life Sciences, Hebei University, Baoding, 071002, P. R. China

**Keywords:** Coleoptera, Tenebrionidae, Lagriinae, *Laena*, new species, China, Xizang, Yunnan, Hainan Island

## Abstract

Six new species of *Laena* Dejean, *Laena quadrata* **sp. n.** and *Laena motogana* **sp. n.**(China: Xizang), *Laena chiloriluxa* **sp. n.**, *Laena dentata* **sp. n.** and *Laena liangi* **sp. n.** (China: Yunnan), *Laena dentatocrassa* **sp. n.** (China: Hainan Island, representing new province record of the genus) are described, complemented with photos of habitus, illustrations of legs, antenna, aedeagus and last abdominal ventrite of male and female. Type specimens are deposited in both the Museum of Hebei University, Baoding, China and the Natural History Museum of Stuttgart, Germany.A key to the 102 Chinese species of genus *Laena* is provided.

## Introduction

Until now about 330 species of the genus *Laena* Dejean were described from the Palaearctic and Oriental Regions, from which about 105 species (including the six new ones described below) were found in China. Most of them were described by the following authors: Schuster (1916, 1940), Kaszab (1956, 1970), Li and Wang (1993), Masumoto and Yin (1993, 1994), Masumoto (1996, 1998), Ren and Hua (2006), Schawaller (2001, 2008) and Zhao and Ren (2011).

Recently six new species of *Laena* were identified from Xizang, Yunnan and Hainan Island (representing new province record of the genus) of China. The collecting localities of these new species are depicted in Fig. 58.

## Taxonomy

### Laena

Dejean, 1821

Laena Dejean, 1821: 64; Latreille, 1829: 39.
Psilolaena Helier, 1923: 70.
Catolaena Reitter, 1900: 282.
Ebertius Jedlička, 1965: 98.

#### Type species.

*Scaurus viennensis* J. Sturm, 1807.

#### Key to the species of *Laena* in China

**Table:** 

1	Species from the southeastern and south provinces Guizhou, Guangxi, Jiangxi, Fujian and Hainan	2
–	Speicies from the western and central provinces Xizang (=Tibet), Yunnan, Sichuan, Shaanxi, Hubei, Gansu and Henan	8
2	All femora with distinct teeth	3
–	All femora without armature	5
3	All femora each with a tooth	*Laena dentatocrassa* sp. n.
–	All femora each with a pair of unequal teeth	4
4	Lateral margins of pronotum unbordered	*Laena hlavaci*
–	Lateral margins of pronotum bordered	*Laena cooteri*
5	Basal margin of pronotum nearly as wide as distal margin	*Laena guangxica*
–	Basal margin of pronotum distinctly narrower than distal margin	6
6	Pronotum widest in the middle	*Laena guizhouica*
–	Pronotum widest anteriorly	7
7	Small body size (5.5–6.0 mm); aedeagus with triangular apicale	*Laena jiangxica*
–	Large body size (8.0–8.5 mm); aedeagus with rounder tip of apicale	*Laena fanjingshanana*
8	Pronotum with distinctly protruding anterior corners, thus anterior pronotal margin with distinct convex excavation	9
–	Pronotum without protruding anterior corners, anterior pronotal margin straight or at most weakly excavated	12
9	Lateral margins of pronotum unbordered	*Laena quinquagesima*
–	Lateral margins of pronotum bordered	10
10	Pronotal surface with distinct impressions, elytral intervals flat with scattered fine punctures	*Laena basumtsoica*
–	Pronotal surface flat, without impressions, elytral intervals wrinkled with large and nearly confluent punctures	11
11	Body length 7.0 mm, male posterior tibiae interiorly without granules, aedeagus with broad, spade-like apicale	*Laena janatai*
–	Body length 6.0–6.5 mm, male posterior tibiae interiorly with granules, aedeagus with longer apicale with knob-like tip	*Laena michaeli*
12	Elytral interval VII keel-like and pronounced, lateral intervals VIII and IX not or indistinctly visible in dorsal view, internal intervals between these keel-like intervals flat or nearly flat	13
–	Elytral interval VII not keel-like, sometimes interval VII distinctly convex or sometimes intervals III, V and VII convex, but interval VII not separating the joint elytra in a flat interior part and a vertical lateral part	23
13	All femora in both sexes with distinct teeth or angles	14
–	All femora in both sexes completely without armature	15
14	All femora with distinct angles but without teeth, lateral margins of pronotum unbordered and rounded	*Laena kubani*
–	All femora with distinct teeth, lateral margins of pronotum bordered	*Laena dabashanica*
15	Elytral interval VII keel-like, swollen and knob-like in the humeral region	16
–	Elytral interval VII keel-like over its total length and not swollen in the humeral region	18
16	Joint elytra about twice as long as wide; elytra with rows of fine punctures extinguishing in the posterior part; elytral intervals dull and without setation; aedeagus with long apicale	*Laena mirabilis*
–	Joint elytra about 1.6 times as long as wide; elytra with rows of large punctures; elytral intervals shining and with adpressed setation; aedeagus with broad apicale	17
17	Body length 8.8–9.5 mm; elytral intervals wrinkled and with dense and coarse punctation; posterior tibiae of males armed with spines	*Laena mulica*
–	Body length 7.0–7.5 mm; elytral intervals flat with fine punctation; posterior tibiae of males without spines	*Laena maowenica*
18	Body length 4.7–5.2 mm; lateral margins of pronotum crenulated	*Laena yajiangica*
–	Body length over 6.0 mm; lateral margins of pronotum smooth	19
19	Pronotum besides impressions flat, so disc on the same level as lateral margins	20
–	Pronotum besides impressions more or less convex, so disc higher than lateral margins	21
20	Base of pronotum with distinct impression (besides other impressions), this base distinctly narrower than anterior margin with protruding anterior corners; apicale of aedeagus broad, spade-like	*Laena yulongica*
–	Base of pronotum without distinct impression, this base about as wide as anterior margin with rounded anterior corners; apicale of aedeagus longer, triangular	*Laena bowaica*
21	Pronotum and elytra shining; pronotum strongly convex with distinct lateral border, basal margin bent downwards; elytra rows with fine punctures	*Laena haigouica*
–	Pronotum and elytra dull; pronotum feebly convex with marked but unbordered lateral margins, basal margin not bent downwards; elytral rows with large punctures	22
22	Base of pronotum distinctly narrower than anterior margin; punctures of elytral intervals as large as puntures of the rows; apicale of aedeagus triangular	*Laena habashanica*
–	Base of pronotum about as wide as anterior margin; punctures of elytral intervals distinctly smaller than puntures of the rows; apicale of aedeagus spade-like	*Laena schuelkei*
23	All femora or at least anterior femora in both sexes medially with teeth or distinct angles	24
–	All femora without distinct modifications	53
24	Elytra (not pronotum) without any setation in the elytral rows and intervals	25
–	Elytra with long erect or short adpressed setae in the elytral rows and or in the elytral intervals	32
25	Only anterior femora with distinct angles, middle and posterior femora without armature	*Laena jinpingica*
–	All femora with distinct teeth	26
26	Posterior tibiae of males without distinct modifications	27
–	Posterior tibiae of males with modifications (hooked interior apex or medially with tooth or dilatation)	30
27	Lateral margins of pronotum unbordered	28
–	Lateral margins of pronotum bordered	29
28	Body length 6.2–7.5 mm, aedeagus with longer apicale with rounded finger-like tip	*Laena smetanai*
–	Body length 7.5–8.5 mm, aedeagus with broad apicale with blunt tip	*Laena bohrni*
29	Basal margin of pronotum distinctly narrower than distal margin, pronotum cordiform; aedeagus with longer triangular apicale; anterior femora of males of similar size as middle and posterior femora, anterior femora besides hook-like tooth with rounded anterior corner	*Laena chinensis*
–	Basal margin of pronotum as wide as distal margin, pronotum subquadrate; aedeagus with broader spade-like apical; anterior femora of males extraordinary thick and besides broad tooth with additional tooth-like anterior corner	*Laena sehnali*
30	Posterior tibiae of males with distinctly hooked inner apex, medial side with granules; apicale of aedeagus about twice as long as wide	*Laena turnai*
–	Posterior tibiae of males medially dilatated or with teeth, without granules; apicale of aedeagus subquadrate	31
31	Elytral inervals with distinct punctation; male anterior tibiae with parallel inner side; male posterior tibiae medially with tooth	*Laena tabanai*
–	Elytral inervals only with very fine and scattered punctures; male anterior tibiae medially with dilatation; male posterior tibiae medially with rounded dilatation	*Laena hubeica*
32	Only anterior femora with teeth, medial and posterior tibiae without modifications	33
–	All femora with teeth or at least with distinct angles	34
33	Anterior tibiae of males with parallel-sided broadened anterior part; posterior tibiae of males without hooked inner apex	*Laena quadrata* sp. n.
–	Anterior tibiae of males without modifications; posterior tibiae of males with hooked inner apex	*Laena yasuakii*
34	Male dorsal side with green metallic shine	*Laena chiloriluxa* sp. n.
–	Male dorsal side without green metallic shine	35
35	Middle femora each with a pair of teeth	*Laena dentata* sp. n.
–	Middle femora each with a tooth	36
36	Body length 9.3–9.6 mm; elytra with dense and large punctation of the intervals, so the elytral intervals are indistinct	*Laena shaluica*
–	Body length less than 8.0 mm; elytra with distinct elytral rows and separated intervals	37
37	Lateral margins of pronotum distinctly bordered	38
–	Lateral margins of pronotum unbordered, sometimes feebly marked	41
38	Eyes prominent; pronotum cordiform with the basal margin distinctly narrower than anterior margin; body length 6.6–7.9 mm	*Laena angulifemoralis*
–	Eyes not prominent; pronotum subquadrate with the basal margin as wide as the anterior margin; body length 3.5–6.0 mm	39
39	Pronotum and elytra with erect and long setation; apicale of aedeagus short and broad	*Laena luguica*
–	Pronotum and elytra with short and adpressed setation; apicale of aedeagus long and triangular	40
40	Pronotum widest in the middle	*Laena formaneki*
–	Pronotum widest near the anterior corners	*Laena farkaci*
41	Pronotum with coarse, partly confluent punctation, surface of pronotum wrinkled or uneven	42
–	Pronotum with fine punctation, punctures always distinctly separated, surface of pronotum smooth	45
42	Odd-numbered elytral intervals distinctly convex; all femora with distinct teeth	*Laena ganzica*
–	All elytral intervals homogeneous, either all slightly convex or all flat; femora only with angles, the latter partly reduced	43
43	Pronotum subquadrate with the basal margin as wide as the anterior margin; male anterior tibiae medially with distinct tooth	*Laena ludingca*
–	Pronotum cordiform with the basal margin narrower than the anterior margin; male anterior tibiae without modification	44
44	Elytral intervals densely scattered with large and confluent punctures; aedeagus with broad apical with blunt tip	*Laena jizushana*
–	Elytral intervals with a row of separated small punctures; aedeagus with triangular apicale	*Laena yufengsi*
45	Pronotum and elytra with dull shagreened surface and with long, dense and erect setation	*Laena qinlingica*
–	Pronotum and elytra with shining surface and with sparser short and adpressed setation	46
46	Pronotum with fine and sparse punctation; pronotal punctures distinctly smaller than punctures of the elytral rows	*Laena lisuorum*
–	Pronotum with large punctures, which are of similar size as those of the elytral rows	47
47	Pronotum cordiform, basal margin distinctly narrower than anterior margin	48
–	Pronotum either subquadrate or trapezoid, basal margin as wide as or only slightly narrower than anterior margin	49
48	Joint elytra oval; aedeagus with longer triangular apicale	*Laena wolongica*
–	Joint elytra longer and parallel; aedeagus with broad apicale with blunt tip	*Laena barkamica*
49	Pronotum widest in the middle	50
–	Pronotum widest before the middle (three quite similar species, compare body length, dorsal punctation, setation and shape of aedeagus and distribution)	51
50	Body length 3.7–3.8 mm; posterior tibiae of males only with finely hooked interior apex; aedeagus with longer triangular apicale	*Laena daxueica*
–	Body length 5.0 mm; posterior tibiae of males with finely hooked interior apex and medially swollen in the middle; aedeagus with broader apicale with blunt apex	*Laena cylindrica*
51	Body length above 5.5 mm. –Shaanxi and N Sichuan	*Laena fengileana*
–	Body length 3.0–4.2 mm. –S Tibet and Yunnan	52
52	Punctures of elytral intervals smaller than pronotal punctures; punctures of elytral rows without setae, only elytral intervals with setae. –S Tibet	*Laena gracilis*
–	Punctures of elytral rows equal in size to pronotal punctures, each puncture bearing a seta, intervals with a row of fine punctures bearing a similar seta. –Yunnan	*Laena septuagesima*
53	Small species (4.2–6.0 mm) with distinctly dull surface and pronotum with coarse and confluent punctation	54
–	Small and large species; if body length below 6.0 mm then with shining surface and pronotum with separated punctation	55
54	Elytral intervals III, V and VII distinctly but equally convex, other elytral intervals flat; lateral margins of pronotum marked and crenulated but unbordered; aedeagus with longer triangular apicale	*Laena davidi*
–	Elytral intervals III and V slightly, VII distinctly convex, intervals with rows of distinct granules; lateral margins of pronotum not bordered and not marked; aedeagus with broad spade-like apicale	*Laena gaoligongica*
55	Elytra (not pronotum) without any setation or with very short setation in the elytral rows and or intervals (setae not distinctly longer than a diameter of the punctures in the rows)	56
–	Elytra with distinct adpressed shorter or erect longer setation in the elytral rows and/or intervals	84
56	Punctation of elytra confused, surface not distinctly separated in elytral rows and intervals	57
–	Elytra always with distinct elytral rows and punctate or impunctate intervals, sometimes punctures in the intervals as large as in the rows, sometimes the elytral rows extinguished in the posterior part of the elytra	59
57	Body length 5.5–6.2 mm; punctation on pronotum and elytra distinctly separated, surface shining	*Laena heinzi*
–	Body length 6.5–8.0 mm; punctation on pronotum and elytra confluent, surface dull	58
58	Pronotum distinctly broader than long; elytra 1.5 times as long as wide, widest in posterior third	*Laena businskyorum*
–	Pronotum nearly as wide as long; elytra 1.8 times as long as wide, widest in the middle; few differences, but no intermediate forms known	*Laena deqenica*
59	Elytral intervals besides the puncture rows with distinct punctation, these punctures densely scattered and about half as large as punctures in the rows	*Laena safraneki*
–	Elytral intervals between the puncture rows without distinct punctation or only with an indistinct row of very fine punctures	60
60	Small species (4.4–6.6 mm) from Tibet with a flat and subquadrate pronotum, pronotal disc smooth and without impressions, lateral pronotal margin bordered	61
–	Smaller and larger species with different structure of the pronotum; if with similar pronotum then not from Tibet, if from Tibet then with a different pronotum	64
61	Male tibiae without secondary sexual characters	62
–	Anterior and/or posterior male tibiae with secondary sexual characters	63
62	Pronotum with rounded lateral margins; elytral punctures larger, distance sometimes only about 1 diameter	*Laena alticola*
–	Pronotum with parallel lateral margins; elytral punctures finer, distance always over 2 diameter; few differences, but no intermediate forms known	*Laena parallelocollis*
63	Anterior and posterior tibiae of males swollen medially; apicale of aedeagus broad with blunt tip	*Laena tuntalica*
–	Anterior tibiae of males slightly excavated medially; apicale of aedeagus narrower and triangular	*Laena cholanica*
64	Large species (7.8–10.3 mm) with long and parallel elytra, with a flat subquadrate pronotum with bordered lateral margins, and with a row of distinct spines interiorly in the distal half of the male posterior tibiae	65
–	Larger or smaller species with a different combination of these characters	68
65	Pronotum about 1.1–1.3 times as broad as long; apicale of aedeagus broad and with blunt tip	66
–	Pronotum about as wide as long; apicale of aedeagus triangular	67
66	Pronotum widest before the middle; male anterior tibiae without modification	*Laena tryznai*
–	Pronotum widest behind the middle; male anterior tibiae excavated medially	*Laena kalabi*
67	Pronotum with parallel sides; posterior tibiae of males besides spines distally somewhat swollen but wihtout distinctly hooked inner apex	*Laena dickorei*
–	Pronotum with rounded sides; posterior tibiae of males besides spines distally somewhat swollen and with distinctly hooked inner apex	*Laena gyamdaica*
68	Lateral margins of pronotum completely unbordered and also not marked	69
–	Lateral margins of pronotum completely or at least partly bordered	71
69	Body small-sized (3.8–4.8 mm); pronotum cordiform, basal margin distinctly narrower than anterior margin	*Laena diancangica*
–	Body medium-sized (6.5–8.8 mm); pronotum round	70
70	Anterior tibiae of males medially with a distinct tooth; joint elytra of oval shape	*Laena schusteri*
–	Anterior tibiae of males without modification; joint elytra long and parallel	*Laena hengduanica*
71	Posterior tibiae of males medially with a distinct hump-like dilatation shortly before apex	*Laena baishuica*
–	Posterior tibiae of males with different modification or without secondary sexual characters	72
72	Posterior tibiae of males medially with a single spine shortly before apex	*Laena naxiorum*
–	Posterior tibiae of males with different modification or without secondary sexual characters	73
73	All tibiae in male without distinct modifications	74
–	Anterior and/or posterior tibiae in male modified	78
74	Pronotum cordiform, basal margin of pronotum distinctly narrower than anterior margin	75
–	Pronotum broad, subquadrate or round, basal margin not distinctly narrower than anterior margin	76
75	Body length 4.6 mm; aedeagus with long triangular apicale	*Laena zogqenica*
–	Body length 6.0–8.5 mm; aedeagus with spade-like apicale with blunt tip	*Laena fouquei*
76	Pronotum flat and subquadrate	77
–	Pronotum convex and round	*Laena langmusica*
77	Pronotum with large but sparse punctation; aedeagus with triangular apicale with acute tip	*Laena alesi*
–	Pronotum with fine but dense punctation; aedeagus with spade-like apicale with blunt tip	*Laena nyingchica*
78	Elytral rows distinctly extinguished in the posterior part of the elytra; elytral intervals distinctly shagreened and dull	*Laena xuerensis*
–	Elytral rows more or less complete; elytral intervals shining	79
79	Pronotum cordiform, its base distinctly narrower than anterior margin	80
–	Pronotum round or subquadrate, its base more or less as wide as anterior margin	81
80	Body length 10.0–11.5 mm; elytra with rows of punctures in distinct striae; posterior tibiae of males swollen interiorly in the middle; apicale of aedeagus thin and finger-like	*Laena gigantea*
–	Body length 8.0–9.0 mm; elytra with rows of punctures without striae; posterior tibiae of males swollen interiorly at base; apicale of aedeagus broad spade-like	*Laena baiorum*
81	Basal margin of pronotum bent downwards, so this margin is on a distinctly deeper lever than disc	*Laena kangdingica*
–	Basal margin of pronotum not bent downwards, so this margin is more or less on the same level as disc	82
82	All tibiae of males with a few indistinct granules at the inner side, but without excavations or dilatations; body length in the average smaller (4.5–6.5 mm)	*Laena xueshanica*
–	Anterior tibiae of males interiorly with excavation, posterior tibiae interiorly swollen or with hooked apex	83
83	Pronotum round; posterior tibiae of males interiorly with hooked apex; apicale of aedeagus longer spade-like	*Laena zongdianica*
–	Pronotum subquadrate; posterior tibiae of males interiorly with dilatation in the distal part; apicale of aedeagus very short and broad	*Laena nujiangica*
84	Eyes prominent; posterior tibiae of males medially swollen and interiorly with hooked apex	*Laena baoshanica*
–	Eyes not prominent; posterior tibiae of males either differently modified or completely unmodified	85
85	Elytral intervals between elytral rows either with distinct scattered punctures or with an additional row of large punctures (interval punctures about half as large as punctures of the rows)	86
–	Elytral intervals without or only with a row of indistinct very fine punctures in the elytral intervals	89
86	Elytral intervals with a single row of distinct punctures; posterior tibiae of males medially granulated	*Laena becvari*
–	Elytral intervals with scattered dense and large punctation	87
87	Posterior tibiae of males medially with a distinct tooth; apicale of aedeagus broad and spade-like	*Laena houzhenzica*
–	Posterior tibiae of males only with finely hooked inner apex; apicale of aedeagus longer and triangular	88
88	Pronotum and elytra dull, punctation of pronotum confluent; anterior femora of males medially granulated; anterior tibiae of males with a hooked inner apex	*Laena bifoveolata*
–	Pronotum and elytra shining, punctation of pronotum separated; anterior femora of males smooth; anterior tibiae of males distinctly swollen medially	*Laena puetzi*
89	Pronotum and elytra with long and erect setae	90
–	Pronotum and elytra with short, adpressed setae	96
90	Pronotum round or cordiform	91
–	Pronotum subquadrate or trapezoid	92
91	Pronotum round; base of pronotum indistinctly bordered and bent downwards; pronotum with fine punctation, these punctures distinctly finer than punctures in the elytral rows	*Laena hingstoni*
–	Pronotum cordiform; base of pronotum unbordered and not bent downwards; punctures of pronotun as large as those of elytral rows	*Laena motogana* sp. n.
92	Pronotum trapezoid	93
–	Pronotum subquadrate	94
93	All tibiae of males without modification	*Laena moxica*
–	Middle and posterior tibiae of males with hooked inner apex, posterior tibiae of males with small spines medially	*Laena zhengi*
94	All tibiae of males with granulation at apical half of inner sides	*Laena liangi* sp. n.
–	All tibiae of males without modification	95
95	Apicale of aedeagus triangular with sinuated lateral margins and finger-like tip	*Laena watanabei*
–	Apicale of aedeagus broad with blunt tip	*Laena daliensis*
96	Pronotum with coarse punctation, punctures often confluent, disc with distinct impressions, surface dull and shagreened	97
–	Pronotum with finer punctation, punctures never confluent, disc without impressions, surface shining	99
97	Anterior margin of pronotum excavated and anterior corners protruding, lateral margins distinctly bordered	*Laena yuzhuensis*
–	Anterior margin of pronotum not distinctly protruding, lateral margins unbordered or at least indistinctly marked	98
98	Elytral inner intervals flat, intervals V and VII convex; intervals about 3 times as wide as the punctures of the elytral rows; setae of elytra intervals distinctly longer than setae of the rows	*Laena luhuoica*
–	Elytral intervals equally convex; intervals about 1–2 times as wide as the punctures of the elytral rows; setae of elytral intervals as long as setae of the rows	*Laena wrasei*
99	Pronotum widest in the anterior third, lateral margins bordered	100
–	Pronotum widest in the middle, lateral margins unbordered	101
100	Elytral intervals with a row of fine punctures bearing short adpressed setae; elytra longer and parallel. –Sichuan	*Laena paomaica*
–	Elytral intervals scattered with dense fine punctation, punctures bearing longer adpressed setae; elytra shorter and oval.–Yunnan	*Laena gyalthangica*
101	Apicale of aedeagus triangular with straight sides and with acute tip	*Laena brendelli*
–	Apicale of aedeagus broad with sinuated sides and blunt tip	*Laena emeishana*

### Laena quadrata sp. n.

urn:lsid:zoobank.org:act:10B63410-9E19-436F-8E04-E4D137BBF94B

http://species-id.net/wiki/Laena_quadrata

Figs 1 7–14

#### Type material.

Holotype ♂ (MHBU): China, Xizang, Gyaca Coun., Lasui [29.0649°N, 92.4656°E], 3500 m, 29 June 2009, G. D. Ren leg.

#### Etymology.

Named after the shape of the pronotum.

#### Diagnosis.

The new species shares with *Laena yasuakii* Masumoto, 1996 from Yunnan having the profemora with tooth, but it can be separated mainly by the shape of the pronotum and aedeagus, protibiae with a parallel-sided broadened anterior part.

**Figures 1–6.**
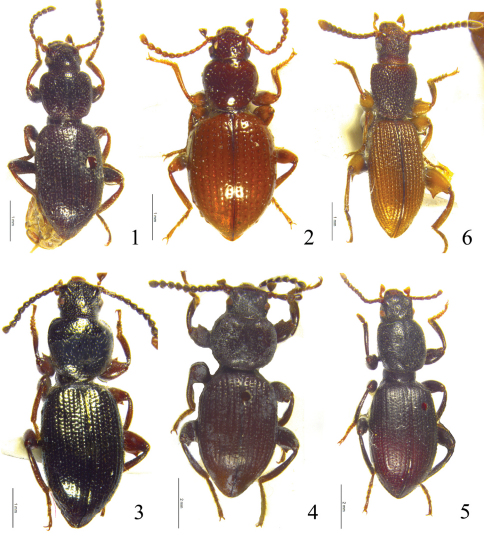
**1** *Laena quadrata* sp. n., male **2** *Laena motogana* sp. n., male **3** *Laena chiloriluxa* sp. n., male **4** *Laena dentata* sp. n., male **5** *Laena liangi* sp. n., male **6** *Laena dentatocrassa* sp. n., male**.**

#### Description.

**Figures 7–14.**
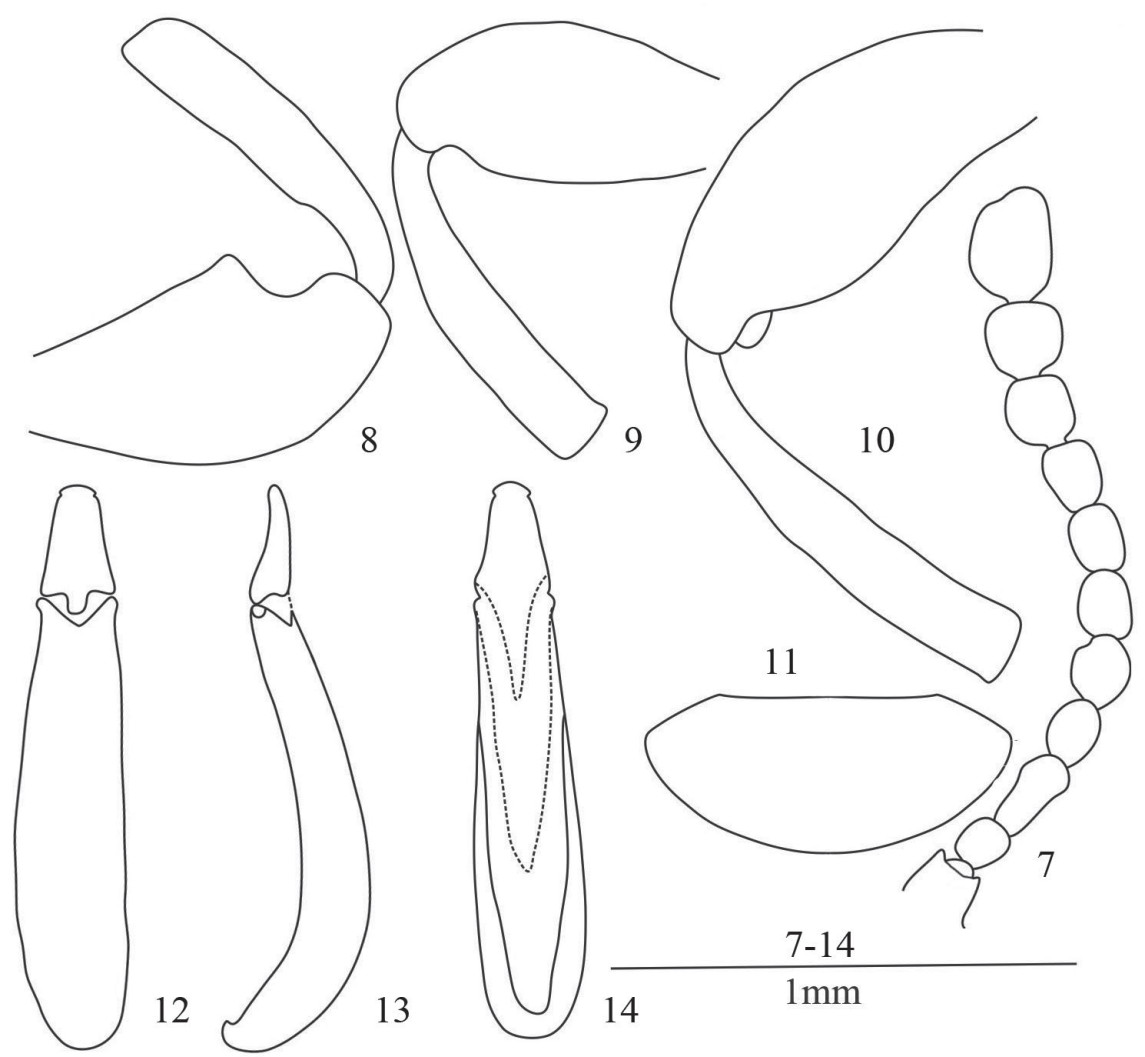
*Laena quadrata* sp. n. **7** male antenna, dorsal view **8** anterior femur and tibia, male, ventral view **9** middle femur and tibia, male, ventral view **10** posterior femur and tibia, male, ventral view **11** last abdominal ventrite, male, ventral view **12–14** aedeagus in dorsal, lateral and ventral views.

Male. Eyes (Fig. 1) elliptical, moderately prominent. Antennae (Fig. 7) extending to base of pronotum, ratio of length (width) of antennomeres II–XI as follows: 5.5 (4.9): 10.0 (4.8): 7.1 (5.5): 7.9 (6.5): 7.1 (6.0): 7.3 (5.9): 7.3 (6.1): 8.0 (7.5): 8.5 (8.3): 12.5 (9.1).

Pronotum (Fig. 1) nearly quadrate, 1.2 times as wide as long, widest at middle; disc with large punctures, punctures medially somewhat sparser than laterally, their distance 1–4 times as long as puncture diameter, most punctures with long and erect setae, surface flat and shining, lateral margins weakly bordered, basal margin unbordered and not bent downwards, posterior angles rounded; propleura with smaller and sparser punctures and shorter setae than those of disc.

Elytra (Fig. 1) nearly parallel-sided from base to middle, 1.9 times as long as wide, widest at middle; punctural rows without striae, punctures as large as those of pronotum, each puncture with long and erect seta, intervals with regular row of small punctures each bearing a similar seta, all intervals flat and shagreened, interval IX with 4 indistinct setiferous umbilicate pores, interval VII without them.

Anterior femur (Fig. 8) with tooth, other femora (Figs. 9–10) unarmed. Anterior tibiae with parallel-sided broadened anterior part.

Last abdominal ventrite (Fig. 11) rounded at apex. Aedeagus see Figs. 12–14.

Female: unkown.

Body length: 4.5 mm.

### Laena motogana sp. n.

urn:lsid:zoobank.org:act:C8F21615-30DE-49A1-AB8B-A159204CAD47

http://species-id.net/wiki/Laena_motogana

Figs 2 15–22

#### Type material.

Holotype ♂ (MHUB): China, Xizang, Môdog Coun., Nage-Dayan dong, 2900–3300 m, 12 August 2005, L. Tang leg.

Paratype: 1♂ (MHUB): labelled as the holotype; 1♂, (MHUB): China, Xizang, Môdog Coun., Hanmi, 2200 m, 23–27 August 2005, L. Tang leg; 1♂ (SMNS): China, Xizang, Môdog Coun., Nage, 3000–3500 m, 11 August 2005, L. Tang leg.

#### Etymology.

Named after the type locality.

#### Diagnosis.

The new species is similar to *Laena hingstoni* Schuster, 1926, both from Xizang, by similar body shape, but can be separated by the smaller body size, the completely unbordered lateral margin of the pronotum, and flat elytral intervals.

#### Description.

**Figures 15–22.**
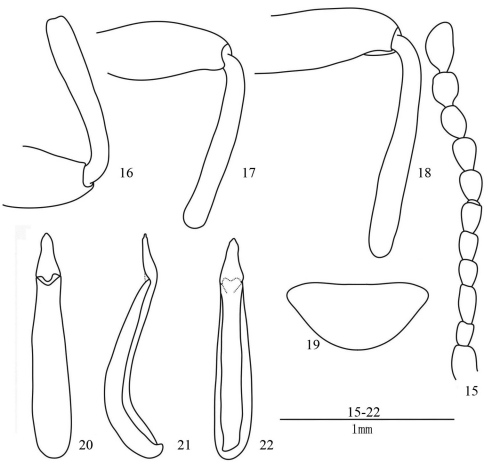
*Laena motogana* sp. n. **15** male antenna, dorsal view **16** anterior femur and tibia, male, ventral view **17** middle femur and tibia, male, ventral view **18** posterior femur and tibia, male, ventral view **19** last abdominal ventrite, male, ventral view **20–22** aedeagus in dorsal, lateral and ventral views.

Male. Eyes (Fig. 2) rounded, moderately prominent. Antennae (Fig. 15) extending to base of pronotum, ratio of length (width) of antennomeres II–XI as follows: 7.0 (4.8): 10.8 (5.0): 7.5 (5.3): 8.0 (5.5): 10.0 (5.4): 8.9 (6.3): 9.5 (6.8): 10.0 (7.0): 10.0 (8.0): 14.9 (8.2).

Pronotum (Fig. 2) cordiform, basal margin distinctly narrower than anterior margin, 1.2 times as wide as long, widest just behind anterior margin; disc scattered with some punctures, their distance 2–5 times as long as puncture diameter, all punctures with setae slightly varying in length, surface flat and shining, lateral margins unbordered, basal margin unbordered and not bent downwards, posterior angles rounded; propleura with smaller punctures and shorter setae than those of disc.

Elytra (Fig. 2) oblong ovate, 1.7 times as long as wide, widest at middle; punctural rows without striae, punctures as large as those on pronotum and each bearing a seta, intervals with very fine punctures, each bearing a seta longer than those of punctures of rows, all intervals flat and shining, interval IX with 3 setiferous umbilicate pores.

All femora (Figs. 16–18) without teeth. All tibiae (Figs. 16–18) normal.

Last abdominal ventrite (Fig. 19) triangular at apex. Aedeagus see Figs. 20–22.

Female: unkown.

Body length: 4.3–5.0 mm.

### Laena chiloriluxa sp. n.

urn:lsid:zoobank.org:act:A4C0DDAD-A23A-4C5F-954D-F3B82A789A37

http://species-id.net/wiki/Laena_chiloriluxa

Figs 3 23–31

#### Type material.

Holotype ♂ (MHBU): China, Yunnan Province, Bababhe. N. R. Bengganghan, 1930 m, 14 November 2008, J. Y. Hu & L. Tang leg.

Paratype: 1♂ (SMNS): labelled as the holotype; 1♀ (SMNS), 1♀(MHBU): China, Yunnan, Bababhe, Dianshita, 1900 m, 30 June 2005, LI & LI leg; 1♀ (MHUB): China, Yunnan, Bababhe. N. R. Bengganghan [22.25833° N, 100.66361° E], 1700 m, 14 November 2008, J. Y. Hu & L. Tang leg.

#### Etymology.

Named after the green metallic shine of the body.

#### Diagnosis.

The new species is similar to *Laena luguica* Schawaller, 2001, but can be easily distinguished from it by the following characters: (1) all tibiae of male with finely hooked inner apex, especially the middle tibiae; (2) last abdominal ventrite of male denticulate at apex; (3) the shape of the aedeagus is different.

#### Description.

**Figures 23–31.**
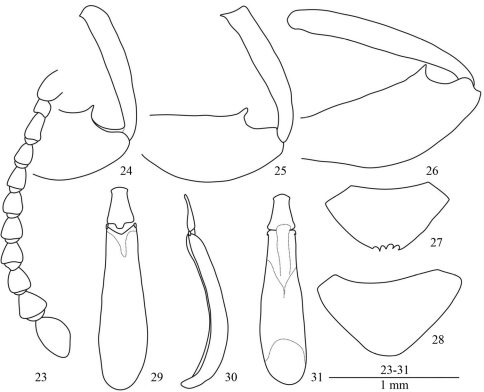
*Laena chiloriluxa* sp. n. **23** male antenna, dorsal view **24** anterior femur and tibia, male, ventral view **25** middle femur and tibia, male, ventral view **26** posterior femur and tibia, male, ventral view **27** last abdominal ventrite, male, ventral view **28** last abdominal ventrite, female, ventral view **29–31** aedeagus in dorsal, lateral and ventral views.

Male. Dorsal side with green metallic shine. Eyes (Fig. 3) elliptical, moderately prominent. Antennae (Fig. 23) extending to base of pronotum, ratio of length (width) of antennomeres II–XI as follows: 5.3 (6.0): 13.3 (6.3): 10.0 (6.7): 10.3 (6.8): 10.1 (7.8): 10.2 (8.1): 10.8 (8.5): 10.0 (8.8): 12.0 (11.5): 18.3 (11.5).

Pronotum (Fig. 3) cordiform, basal margin distinctly narrower than anterior margin, 1.2 times as wide as long, widest just behind anterior margin; disc with small punctures, punctures medially somewhat sparser than laterally, their distance 2–6 times as long as puncture diameter, most punctures with long and erect setae, surface flat and shining, lateral margins bordered, basal margin bordered, feebly in middle, not bent downwards, posterior angles rounded; propleura with smaller punctures and shorter setae than those of disc.

Elytra (Fig. 3) oblong, 2.1 times as long as wide, widest at middle; punctural rows placed in indistinct striae, punctures distinctly larger than those of pronotum and each bearing a long and erect seta, intervals with regular row of small punctures each bearing a similar seta, all intervals flat and shining, interval IX with 5 setiferous umbilicate pores, interval VII in both humeral and posterior region with a setiferous pore.

All femora (Figs. 24–26) each with a strong tooth. All tibiae (Figs. 24–26) with finely hooked inner apex, especially on middle tibiae.

Last abdominal ventrite (Fig. 27) denticulate at apex. Aedeagus see Figs. 29–31.

Female: Dorsal side without green metallic shine. Last abdominal ventrite (Fig. 28) sharp at apex, not denticulate.

Body length: 6.2–7.2 mm.

### Laena dentata sp. n.

urn:lsid:zoobank.org:act:D27A8819-4E08-41AE-AE46-C57D4913B28E

http://species-id.net/wiki/Laena_dentata

Figs 4 32–40

#### Type material. 

Holotype ♂ (MHUB): China, Yunnan, Dali, Cangshan E slope, 3400 m, 19 August 2008, J. S. Xu leg.

Paratype: 1♀ (MHUB): labelled as the holotype.

#### Etymology.

Named after anterior tibiae of male with a medial tooth.

#### Diagnosis.

The new species shares with *Laena schusteri* Schawaller, 2001 the body shape, and the medial tooth of anterior tibia, but can be separated by the teeth of all femora, and middle and posterior tibiae of male with finely hooked inner apex.

#### Description.

**Figures 32–40.**
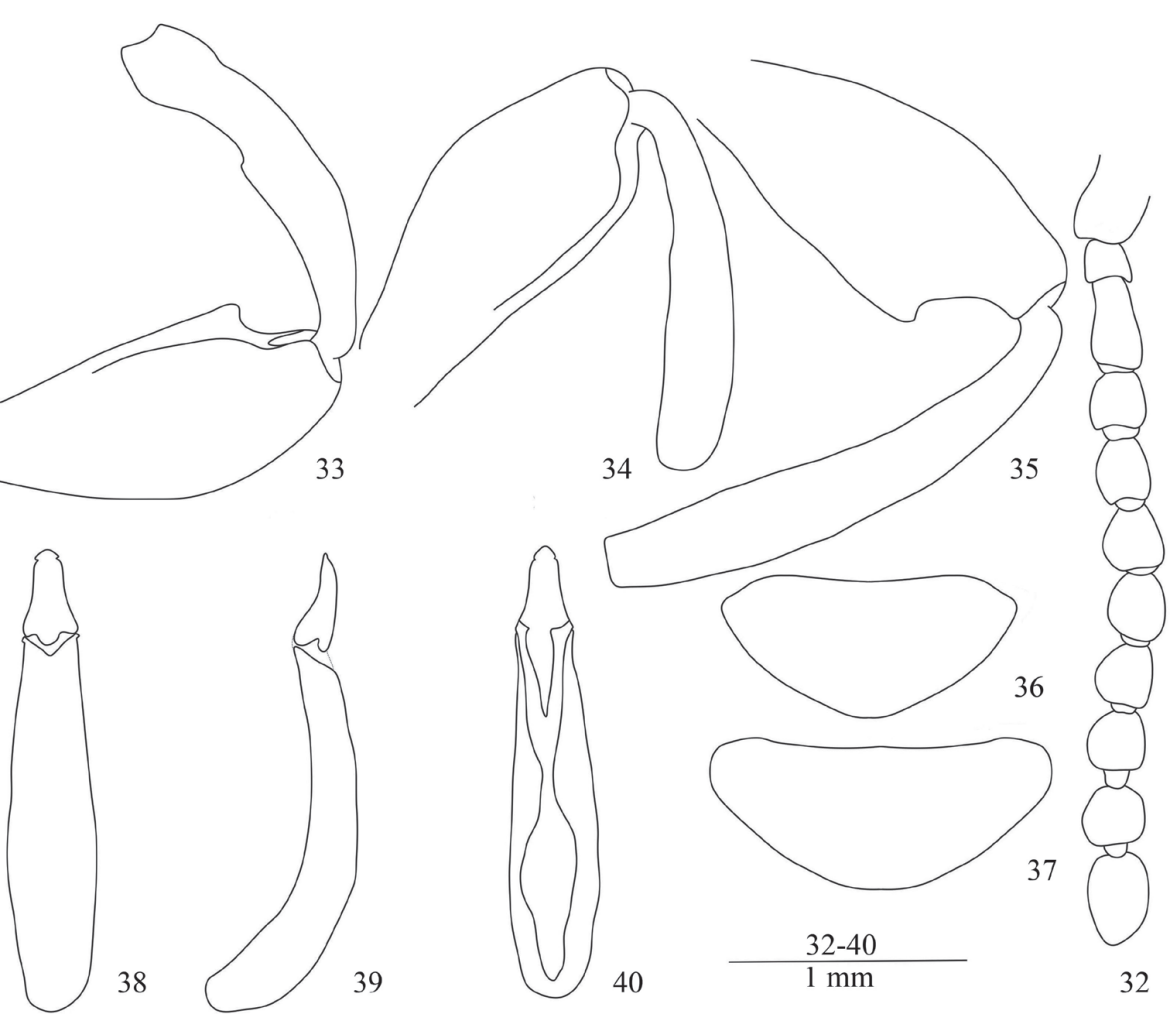
*Laena dentata* sp. n. **32** male antenna, dorsal view **33** anterior femur and tibia, male, ventral view **34** middle femur and tibia, male, ventral view **35** posterior femur and tibia, male, ventral view **36** last abdominal ventrite, male, ventral view **37** last abdominal ventrite, female, ventral view **38–40** aedeagus in dorsal, lateral and ventral views.

Male. Eyes (Fig. 4) elliptical, moderately prominent. Antennae (Fig. 32) extending to base of pronotum, ratio of length (width) of antennomeres II–XI as follows: 7.8 (10.8): 22.1 (11.5): 15.5 (12.8): 16.8 (12.0): 15.0 (13.9): 16.5 (13.9): 15.5 (13.0): 17.8 (13.1): 16.9 (14.2): 20.8 (13.9).

Pronotum (Fig. 4) elliptical, 1.3 times as wide as long, widest at middle; disc with small scattered punctures, their distance 0.5–3 times as long as puncture diameters, all punctures with short and adpressed setae, disc with a pair of feeble impressions, lateral margins narrowly bordered, basal margin unbordered, and not bent downwards, posterior angles rounded; propleura without punctures and setae.

Elytra (Fig. 4) nearly parallel-sided from base to middle, 1.9 times as long as wide, widest at middle; elytra with punctural rows of without striae, punctures distinctly larger than those of pronotum, each puncture bearing a short and adpressed seta, intervals with nearly invisible punctures, but with some similar setae, all intervals flat and shagreened, interval IX with 3 setiferous umbilicate pores.

All femora each with a tooth, but middle femur (Fig. 34) with a pair of equally sized teeth. Anterior tibia (Fig. 33) with median tooth and dilated apex, anterior and middle tibiae excavate medially and with caespitose setae, middle and posterior tibiae (Fig. 35) with finely hooked inner apex.

Last abdominal ventrite (Fig. 36) somewhat sharp at apex. Aedeagus see Figs. 38–40.

Female: Last abdominal ventrite (Fig. 37) somewhat rounded at apex. Middle femora with a pair of unequal teeth.

Body length: 8.5–9.5 mm.

### Laena liangi sp. n.

urn:lsid:zoobank.org:act:1E647A31-054A-4B93-83FF-D93F7764DCD3

http://species-id.net/wiki/Laena_liangi

Figs 5 41–48

#### Type material.

Holotype ♂ (MHUB): China, Yunnan, Gongshan County, No 12 Bridge [27.72°N, 98.60°E], 2750 m, Sino-America Exped, 15 June 2000, H. B. Liang leg.

#### Etymology.

Named after Dr. LIANG Hong-Bin, who collected several new species of *Laena* in China.

#### Diagnosis.

The new species is similar to *Laena kalabi* Schawaller, 2008, but can be easily distinguished from it by the following characters: (1) middle tibiae of male with finely hooked inner apex; (2) anterior and middle tibiae of male medially not sinuate, posterior tibiae of male apex not dilated; (3) shape of the aedeagus is different.

#### Description.

**Figures 41–48.**
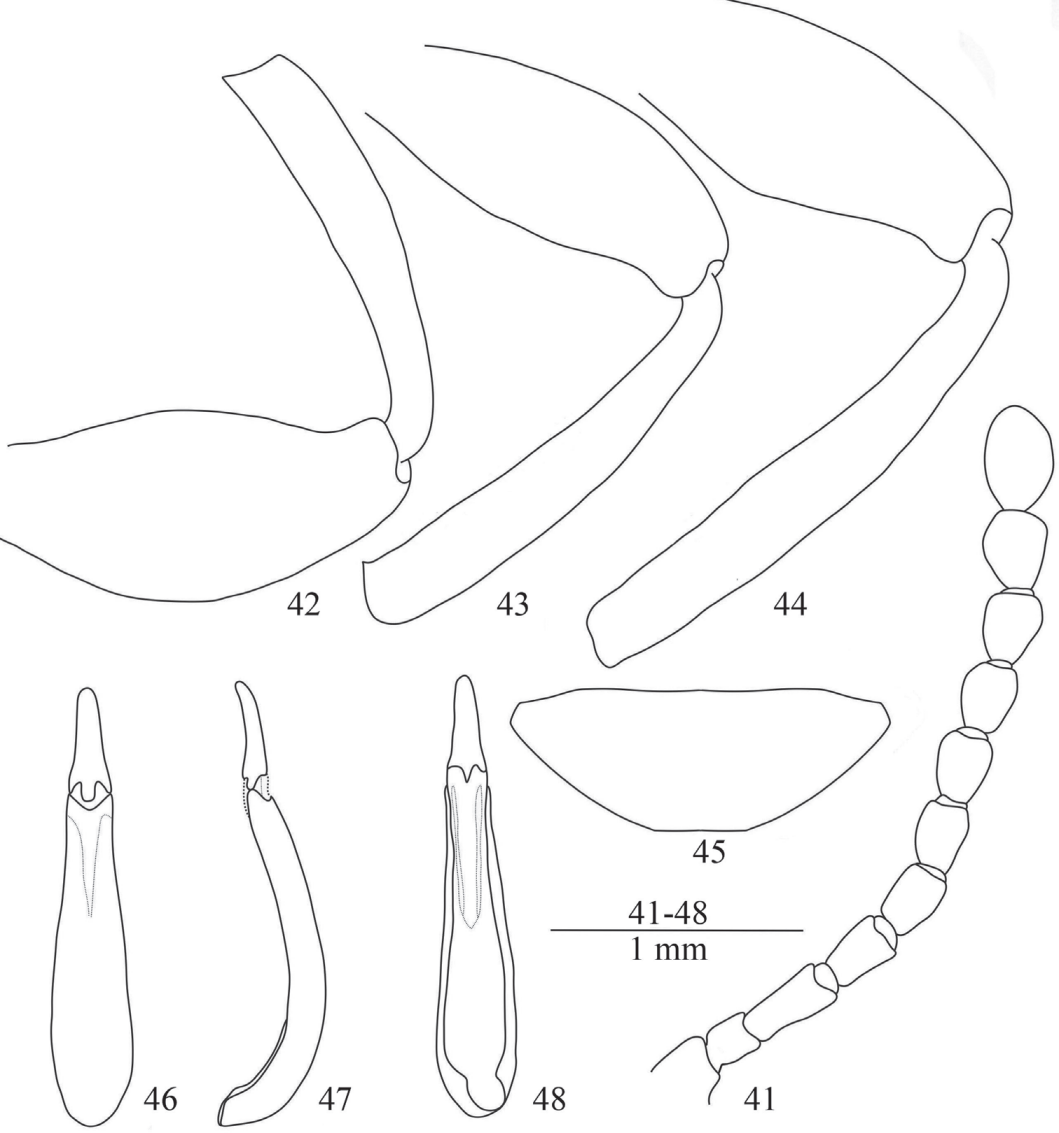
*Laena liangi* sp. n. **41** male antenna, dorsal view **42** anterior femur and tibia, male, ventral view **43** middle femur and tibia, male, ventral view **44** posterior femur and tibia, male, ventral view **45** last abdominal ventrite, male, ventral view **46–48** aedeagus in dorsal, lateral and ventral views.

Male. Eyes (Fig. 5) elliptical, weakly prominent. Antennae (Fig. 41) extending to base of pronotum, ratio of length (width) of antennomeres II–XI as follows: 8.2 (8.5): 21.5 (8.3): 15.5 (8.5): 15.0 (10.0): 14.0 (10.1): 15.5 (11.0): 14.8 (11.0): 15.0 (11.8): 15.9 (13.0): 21.5 (14.0).

Pronotum (Fig. 5) quadrate, 1.0 times as wide as long, widest at middle; disc scattered with small punctures, their distance 1–6 times as long as puncture diameters, all punctures with setae slightly varying in length, surface nearly flat and dull, medial part of base feebly impressied, lateral margins indistinctly bordered, basal margin unbordered and not bent downwards, posterior angles rounded; propleura with larger punctures and shorter setae than those of disc.

Elytra (Fig. 5) nearly parallel-sided, 2.0 times as long as wide, widest at middle; punctural rows in indistinct striae, punctures as large as those on pronotum, and each bearing a shorter seta, intervals with very small punctures, each bearing a similar seta, all intervals flat and dull, interval IX with 3 indistinct setiferous umbilicate pores, interval VII with an indistinct setiferous pore in posterior region.

All femora (Figs. 42–44) without teeth. Middle tibiae (Fig. 43) with finely hooked inner apex, all tibiae (Figs. 42–44) with granulation from middle to apex at inner side.

Last abdominal ventrite (Fig. 45) truncate at apical margin. Aedeagus see Figs. 46–48.

Female: unkown.

Body length: 9.4 mm.

### Laena dentatocrassa sp. n.

urn:lsid:zoobank.org:act:FE8B1802-76E7-4E46-BD42-9D415A5BB39F

http://species-id.net/wiki/Laena_dentatocrassa

Figs 6 49–57

#### Type material.

Holotype ♂ (MHUB): China, Hainan Island, Jianfengling, 25 May 2011, X. Q. Yang & L. F. Wang leg.

Paratype: 1♂ (SMNS), 1♂ (MHUB): China, Hainan Island, Jianfengling, 25 May 2011, X. Q. Yang & C. Zhang leg; 1♂, 2♀♀ (MHUB): labeled as the holotype.

#### Etymology.

Named after the massive teeth of the femora.

#### Diagnosis.

The new species is similar to *Laena jizushana* Masumoto, 1996, but can be easily distinguished from it by the following characters: (1) body with long and erect setae; (2) posterior femur of male with distinct granulation at inner side; (3) all tibiae of male with granulation at inner side and with finely hooked inner apex; (4) the shape of the aedeagus is different.

#### Description.

**Figures 49–57.**
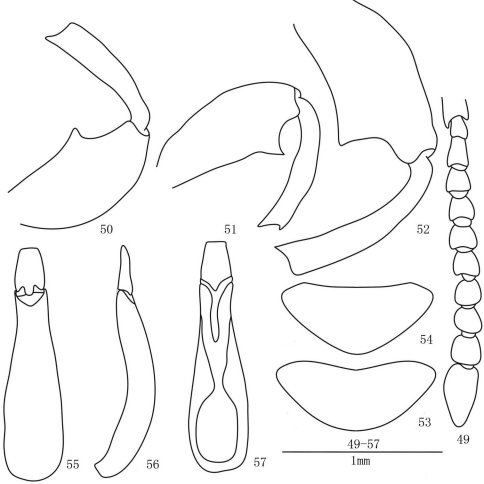
*Laena dentatocrassa* sp. n. **49** male antenna, dorsal view **50** anterior femur and tibia, male, ventral view **51** middle femur and tibia, male, ventral view **52** posterior femur and tibia, male, ventral view **53** last abdominal ventrite, male, ventral view **54** last abdominal ventrite, female, ventral view **55–57** aedeagus in dorsal, lateral and ventral views.

Male. Eyes (Fig. 6) rounded, prominent. Antennae (Fig. 49) extending to base of pronotum, ratio of length (width) of antennomeres II–XI as follows: 5.5 (5.5): 10.8 (6.5): 9.3 (7.5): 8.9 (7.8): 8.3 (9.0): 9.0 (8.5): 8.9 (8.5): 9.0 (8.5): 10.1 (9.8): 19.0 (11.0).

Pronotum (Fig. 6) elongate, 0.9 times as wide as long, widest just behind anterior margin, basal margin distinctly narrower than anterior margin; disc with large punctures, their distance 0.5–2 times as long as puncture diameters, all punctures with long and erect setae, basal part with feeble longitudinal impression, lateral margins indistinctly bordered, basal margin unbordered and not bent downwards, posterior angles rounded; propleura with wider punctures and shorter setae than those of disc.

Elytra (Fig. 6) oblong, 2.4 times as long as wide, widest at middle; elytra punctural rows in indistinct striae, punctures as large as those on pronotum, and each bearing a long and erect seta, intervals with a regular row of small punctures each bearing a similar seta, all intervals convex and shining, interval IX with 10 indistinct setiferous umbilicate pores, interval VII with an indistinct setiferous pore in posterior region.

All femora (Figs. 50–52) each with a strong tooth, posterior femora also with distinct granulation at inner side. All tibiae (Figs. 50–52) with granulation at inner side and with finely hooked inner apex.

Last abdominal ventrite (Fig. 53) nearly rounded at apical margin. Aedeagus see Figs. 55–57.

Females: Ventrite (Fig. 54) nearly sharp at apical margin.

Body length: 5.0–6.0 mm.

**Figure 58.**
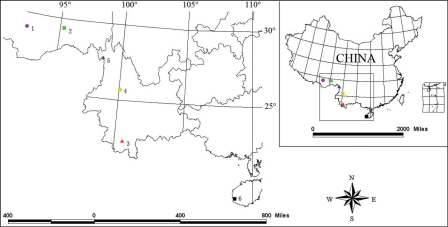
**1** *Laena quadrata* sp. n. **2** *Laena motogana* sp. n. **3** *Laena chiloriluxa* sp. n. **4** *Laena dentata* sp. n. **5** *Laena liangi* sp. n. **6** *Laena dentatocrassa* sp. n.**.**

## Supplementary Material

XML Treatment for Laena

XML Treatment for Laena quadrata

XML Treatment for Laena motogana

XML Treatment for Laena chiloriluxa

XML Treatment for Laena dentata

XML Treatment for Laena liangi

XML Treatment for Laena dentatocrassa
